# Association of aortic stiffness with cognitive decline: Whitehall II longitudinal cohort study

**DOI:** 10.1007/s10654-019-00586-3

**Published:** 2019-11-27

**Authors:** Marzieh Araghi, Martin J. Shipley, Ian B. Wilkinson, Carmel M. McEniery, Carlos A. Valencia-Hernández, Mika Kivimaki, Séverine Sabia, Archana Singh-Manoux, Eric J. Brunner

**Affiliations:** 1 Department of Epidemiology and Public Health, University College London, 1-19 Torrington Place, London, WC1E 6BT UK; 2 Division of Experimental Medicine and Immunotherapeutics, University of Cambridge, Cambridge, UK; 3 Inserm U1153, Epidemiology of Ageing and Neurodegenerative Diseases, Paris, France

**Keywords:** Ageing, Arterial stiffness, Cognitive decline, Pulse-wave velocity

## Abstract

Aortic stiffness is associated with an increased risk of cardio- and cerebrovascular disease and mortality and may increase risk of dementia. The aim of the present study is to examine the association between arterial stiffness and cognitive decline in a large prospective cohort study with three repeated cognitive assessment over 7 years of follow-up. Aortic pulse wave velocity (PWV) was measured among 4300 participants (mean ± standard deviation age 65.1 ± 5.2 years) in 2007–2009 and categorized based on the tertiles: (lowest third: < 7.41 m/s), (middle third: 7.41–8.91 m/s), and (highest third: > 8.91 m/s). A global cognitive score was calculated in 2007–2009, 2012–2013, and 2015–2016 based on responses to memory, reasoning and fluency tests. Standardized global cognitive score (mean = 0, SD = 1) in highest third versus lowest third of PWV category was lower at baseline (− 0.12, 95% CI − 0.18, − 0.06). Accelerated 7-year cognitive decline was observed among individuals with the highest PWV [difference in 7-year cognitive change for highest third versus lowest third PWV: − 0.06, 95% CI − 0.11, − 0.01, *P *< 0.01]. Higher aortic stiffness was associated with faster cognitive decline. Clinicians may be able to use arterial stiffness severity as an indicator to administer prompt treatments to prevent or delay the onset of cognitive decline or dementia. Future studies need to determine whether early intervention of vascular stiffness is effective in delaying these outcomes.

**Electronic supplementary material:**

The online version of this article (10.1007/s10654-019-00586-3) contains supplementary material, which is available to authorized users.

## Introduction

The substantial expansion in life expectancy and population ageing is a major achievement but also one of the 21st century public health challenges. Ageing is associated with exponential increases in number of people living with cognitive impairment and dementia, both of which reduce quality of life [1, 2]. The number of people with dementia is projected to increase from 0.7 M in 2016 to 1.2 M by 2040 in England and Wales [3].

Aortic stiffness, due to loss of elasticity of the artery wall, increases with age and risk factor exposure [4–7] is associated with higher systolic, pulse pressure and atherosclerosis [8], which are in turn important predictors of cardio- and cerebrovascular morbidity and mortality as well as predictors of cognitive decline [9]. The links between cardiovascular disorders and cognitive decline and dementia remain unclear. If such vascular pathophysiological alterations are better understood, it may be possible to prevent or delay cognitive decline and improve quality of life in older people.

Aortic pulse wave velocity (PWV) is the non-invasive gold standard to determine arterial stiffness [10]. Small-scale observational studies (eight cross-sectional studies and five prospective cohort studies) [11–23] have assessed the association of arterial stiffness with cognitive impairment, but their results are inconsistent. It was suggested that arterial stiffness may influence specific cognitive domains such as executive function [11, 20], memory [18, 19, 22], and processing speed [11]. Longitudinal cognitive test data spanning a decade were rare, with small sample size and may have biases attributable to selective attrition (leading to younger persons with better cognitive function over-represented at follow-up). This limits interpretation of previous studies. Further, cognitive function was mainly assessed with the Mini-Mental State Examination (MMSE), a global test with low sensitivity for detection of small changes in cognitive status [24].

Our study is the first longitudinal study with a sensitive cognitive measure (cognitive test battery), large sample size, and high response rate for all three measures of cognitive function (Phases 9, 11, and 12) to examine whether arterial stiffness is associated with deficits and changes in specific cognitive domains. In addition, risk of attrition bias was controlled for using inverse probability weighting to account for missing data over the follow-up period. We hypothesized that higher PWV would be associated with both concurrently assessed cognitive performance and subsequent cognitive decline. We examined a global measure of cognitive function, and the cognitive domains of memory, executive function and verbal fluency using data on 4300 individuals.

## Methods

### Study population

The Whitehall II study is a longitudinal cohort study, initially of 10,308 (67% men, aged 35–55 years) London-based British civil servants, recruited in 1985. Since then, follow-up questionnaire surveys and clinical examinations have taken place every 2–3 years and 5 years, respectively. Ethical approval for each study phase was obtained from the NHS National Research Ethics Service and the local Research Ethics Committee. All participants provided written consent prior to each study phase.

**Fig. 1:**
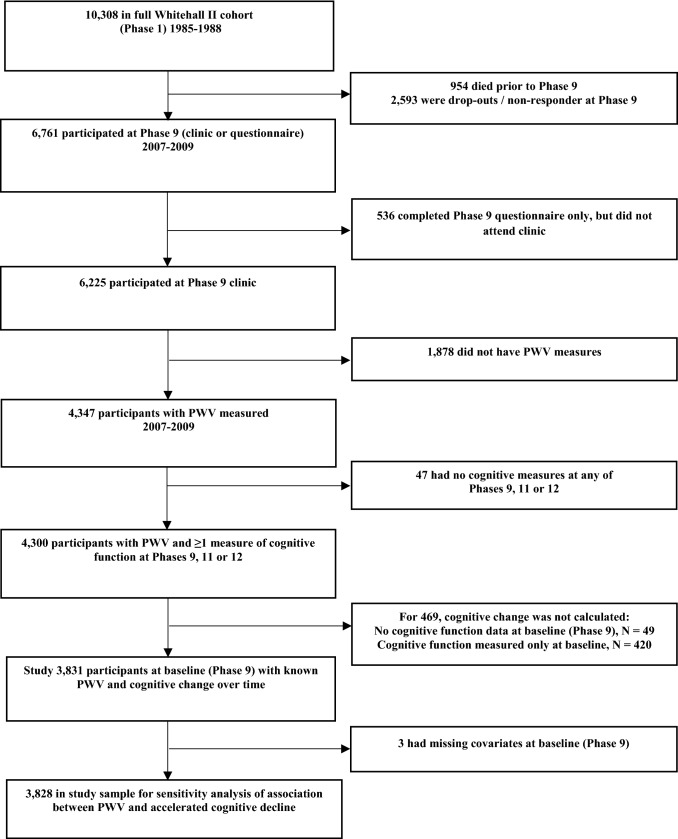
Study flowchart

The 2007–2009 clinical examination was attended by 6225 participants. Of these, 4300 participants provided data on both PWV and cognitive function, forming the analytic sample to examine the association between PWV and cognitive trajectories (Fig. 1). The association between PWV and “pronounced cognitive decline” was assessed in analysis based on 3828 participants.

### Aortic PWV

PWV was assessed between the carotid and femoral sites using applanation tonometry (SphygmoCor, Atcor Medical, Australia), a validated method of measuring PWV. Path length was determined with a tape measure by subtracting the carotid-sternal notch distance from the femoral-sternal notch distance. PWV was measured twice for each participants and a third measurement was taken when the difference between the two measurements were larger than 0.5 m/s. The average of all the measurements was used in the analysis. PWV measurements were repeated within 60 days for 125 participants to assess short-term repeatability. Median difference of the repeats were 0.83 m/s (interquartile range 0.43–1.40).

### Cognitive function

The cognitive test battery was administered at the clinical examinations in 2007–2009, 2012–2013, and 2015–2016. Test–retest reliability was assessed in 556 participants within 3 months and showed excellent reliability (range 0.6–0.9). The cognitive tests were as follows. *Memory:* participants were presented with a 20-word list of one or two syllable words at two second intervals, with 2 min time to write down as many words as they can recall, regardless of word order. *Reasoning:* participants had 10 min to complete the *AH4*-*I* (Alice Heim 4-I), a series of 65 verbal and mathematical reasoning items of increasing difficulty. *Verbal Fluency:* phonemic fluency was assessed via the “S” words and semantic fluency via “animal” words tests. One minute was allowed for each test. To reduce measurement error and ease comparison between tests with different score ranges, we standardized all raw test scores for these four measures to z-scores (mean = 0, standard deviation [SD] = 1) and summed and re-standardized the scores to yield a global cognitive score, as in previous studies [25, 26]. The 30-item Mini-Mental-State-Examination (MMSE) was also used in supplementary analyses, as an alternative measure, to assess global cognitive status.

### Baseline characteristics

Sociodemographic variables included age, sex, ethnicity (white, others), socioeconomic status using employment grade (three categories: high, intermediate, and low representing income and status at work), and education (five categories: less than primary school (up to age 11), lower secondary school (up to age 16), higher secondary school (up to age 18), university, and higher university degree).

Health behaviors were assessed by questionnaire and included smoking (current smokers, ex-smokers, and never smokers) and, alcohol consumption (number of alcoholic drinks consumed in the previous days, converted to units of alcohol consumed in a week and categorized as “no/occasional alcohol consumption,” “moderate alcohol consumption” (1–14 units/week), and “heavy alcohol consumption” (≥ 14 units/week)).

Health related covariates included body mass index assessed at the clinical examination (categorized as < 20, 20–24.9, 25–29.9, and ≥ 30 kg/m^2^), hypertension (systolic/diastolic blood pressure ≥ 140/90 mm Hg or use of antihypertensive drugs), self-reported use of drugs, prevalent diabetes mellitus (determined by fasting glucose ≥ 7.0 mmol/L, reported diabetes diagnosed by a doctor, or use of diabetes drugs), and cardiovascular diseases (CVD) (including coronary heart disease and stroke (identified using linkage to national hospital records)).

### Statistical analysis

Linear mixed models were used to estimate both the cross-sectional and the longitudinal association of PWV with the global cognitive score. These methods use all available cognitive data over the three measures and take into account the fact that repeated measures on the same individual are correlated with each other. The intercept and the slope were fitted as random effects, allowing individuals to have different cognitive scores at baseline and different rates of cognitive decline over the follow-up. The PWV distribution was categorized into thirds: (lowest third: < 7.41 m/s), (middle third: 7.41–8.91 m/s), and (highest third: > 8.91 m/s).

We then fitted the mixed models using these categories of PWV with global cognitive score and also for each cognitive domain as the dependent variable. Since the mean follow-up (time between the first and last cognitive measure) was 7.3 years, we expressed the changes in the cognitive measures per 7 years. The PWV × time × sex interactions suggested no sex differences in the PWV associations with cognitive function or with cognitive decline (all *P *> 0.14) and, therefore, all analyses were conducted with men and women combined. All analyses were adjusted for sex, age, ethnicity and employment grade by fitting these terms and their interactions with follow-up time. Please see the supplementary materials (Model specification) for the full model specification. We also used MMSE < 27 and MMSE < 26 as two ways to define those with cognitive decline and fitted mixed logistic models with these two outcomes, adjusting for age, sex, ethnicity and employment grade, as above. We conducted a sensitivity analysis to assess the potential bias caused by selective attrition over the follow-up period by refitting the models using generalized estimating equations and using inverse probability weights for each observation [27]. The inverse probability weights were obtained by using the 6225 individuals who participated at the Phase 9 clinic and fitting three logistic models to predict which individuals were included in the analysis at Phases 9, 11 and 12 respectively. These logistic models used covariates ascertained at Phase 9 where possible and included sociodemographic factors (age, sex, ethnicity, marital status and employment grade), behavioral factors (smoking status, alcohol consumption and level of physical activity), chronic conditions (coronary heart disease, stroke and diabetes), cardiometabolic risk factors (body mass index, systolic and diastolic blood pressure and serum cholesterol), mental health (General Health Questionnaire caseness) and medication use (anti-hypertensive and lipid lowering).

In order to assess the robustness of the association between PWV and cognition, we examined whether PWV was a predictor of which individuals had the most pronounced cognitive decline. A linear mixed model was first used to estimate the decline in global cognitive score across the three waves of data collection for each participant. A binary outcome variable was then created to indicate the 20% of participants with the fastest cognitive declines compared to those of similar age, sex and employment grade. This was achieved by creating twenty-four 5-year age group by sex by employment grade strata and selecting the 20% of participants with the fastest cognitive declines within each of these strata. Logistic regression models were fitted and odds ratios (ORs) and corresponding 95% confidence intervals (95% CIs) calculated to estimate the association between PWV and the fastest rate of cognitive decline. These models were adjusted for sex, age, ethnicity and employment grade to ensure that there was no residual confounding from these covariates. A sensitivity analysis was conducted to assess the robustness of the estimates to using a different range of thresholds (10–25%) for the proportion of participants with the fastest cognitive decline. Statistical significance was inferred as a two-tailed *P* value < 0.05.

The figure showing the global cognitive score trajectories with time was produced using StataSE 15.1. All other models were fitted using the Proc Mixed, Proc Glimmix and Proc Genmod procedures from the SAS software version 9.4 (SAS Institute, Cary, NC, USA).

## Results

Figure 1 shows the number eligible participants included in the analysis. Of the 10,308 participants at study inception (1985–1988), 954 had died, 2593 had withdrawn from the study, and 536 did not attend clinic in 2007–2009. Our first analysis was based on 4300 of the 6225 individuals still in the study. Those included in the analyses were more educated (40% vs. 32% had a university degree, *P *< 0.001), were younger (65 years vs. 67 years, *P *< 0.001) and generally had a healthier risk factor profile than those excluded (Supplementary Table 1). Demographic and clinical characteristics at baseline among those included in the analyses are shown in Table 1 according to the thirds of PWV. Participants in the highest third were older and had a more adverse risk factor profile than those in the lowest third.

**Table 1:** Baseline characteristics of 4300 participants in the study sample according to the thirds of pulse wave velocity

	[Mean (SD) or %]			*P* value for heterogeneity
	Thirds of baseline pulse wave velocity (m/s)			
	Lowest third (< 7.41)	Middle third (7.41–8.91)	Highest third (> 8.91)	
Number	1436	1438	1426	
Age (y)	63.1 (4.9)	64.8 (5.5)	68.0 (5.6)	< 0.001
Female (%)	28.9	25.1	21.7	< 0.001
Non-white ethnicity (%)	5.6	6.9	10.4	< 0.001
Body mass index (kg/m^2^)				< 0.001
Underweight (< 18.5)	1.1	0.6	0.9	
Normal weight (18.5–24.9)	50.1	37.1	32.7	
Overweight (25.0–29.9)	37.7	47.3	48.2	
Obese (≥ 30.0)	10.4	15.0	18.2	
Systolic blood pressure (mm Hg)	117.6 (13.4)	124.0 (13.9)	132.0 (15.5)	< 0.001
Diastolic blood pressure (mm Hg)	67.5 (9.4)	71.0 (9.5)	73.5 (10.3)	< 0.001
Anti-hypertension medication (%)	23.6	32.9	41.6	< 0.001
Serum cholesterol (mmol/L)	5.28 (1.03)	5.19 (1.04)	5.17 (1.09)	0.009
Lipid lowering medication (%)	23.3	32.0	36.6	< 0.001
Education				< 0.001
≤ Lower secondary	26.4	32.7	36.9	
Higher secondary	29.5	28.1	26.6	
≥ Degree	44.1	39.2	36.5	
Employment grade				0.04
High	51.9	49.3	48.2	
Intermediate	40.7	42.5	41.5	
Low	7.4	8.2	10.3	
Smoking habit				0.03
Never	51.4	47.3	48.6	
Ex-smoker	43.4	47.0	47.5	
Current	5.2	5.8	3.9	
Alcohol consumption				< 0.001
No alcohol	14.5	17.1	17.6	
Moderate alcohol	71.7	65.3	64.2	
Heavy alcohol	13.8	17.6	18.1	
Moderate or vigorous physical activity				0.01
< 1 h/wk	19.3	23.4	23.2	
1–6.9 h/wk	61.7	59.7	61.3	
≥ 7 h/wk	19.0	17.0	15.5	
Diabetes (%)	5.9	9.5	18.5	< 0.001
History of cardiovascular disease (%)	9.6	10.8	14.7	< 0.001

Cross-sectional and longitudinal associations between PWV and global cognitive score and individual cognitive domains, are shown in Table 2. In the cross-sectional analysis, global cognitive score was significantly lower in the middle third and the highest third of PWV (> 8.91 & 7.41–8.91 m/s) compared to the lowest third (< 7.41 m/s). A cross-sectional association was seen for the highest third with all cognitive domains (*P *< 0.05), compared to the lowest third. In the longitudinal analysis, global cognitive function declined significantly faster in the highest third compared to the lowest third after adjustment for age, sex, ethnicity and employment grade [difference in 7-year change in score for > 8.91 m/s versus < 7.41 m/s = − 0.06 (− 0.11 to − 0.01), *P *= 0.01]. Similar effects were seen for some of the cognitive domains but only with phonemic fluency the association was statistically significant (*P *= 0.03). The estimates changed little when the analyses were repeated using inverse probability weights to allow for potential selection bias with the cross-sectional effects being slightly strengthened and the longitudinal effects marginally weakened (Supplementary Table 2). The cross-sectional estimate of the highest third of PWV increased by less than 1% when the 5 prevalent dementia cases were excluded and the longitudinal effect of the highest third of PWV on cognitive function was reduced by 5% when the 10 incident cases of dementia were excluded from the analysis. When participants with a history of CVD were excluded from the analyses, the cross-sectional estimate of the highest third of PWV was reduced by 11% but remained statistically significant (*P *= 0.002), and the longitudinal effect was reduced by 4% and remained statistically significant (*P *= 0.03). When we adjusted for cholesterol levels, the cross-sectional estimate of the highest (vs. lowest) third of PWV decreased by 11% and the longitudinal estimate decreased by 2%.

**Table 2:** Cross-sectional and longitudinal association between the thirds of pulse wave velocity and standardized scores of global cognitive score and individual cognitive domains

Cognitive domain outcome	Pulse wave velocity	Standardized cognitive score at baseline		Change in standardized cognitive score (per 7 years)	
		Difference^a^ (95% CI)	*P* value	Difference^a^ (95% CI)	*P* value
*Global cognitive score*					
	Lowest third	1.0 (Ref)	–	1.0 (Ref)	–
	Middle third	− 0.06 (− 0.12, − 0.01)	0.03	− 0.02 (− 0.06, 0.03)	0.48
	Highest third	− 0.12 (− 0.18, − 0.06)	< 0.001	− 0.06 (− 0.11, − 0.01)	0.01
*Memory*					
	Lowest third	1.0 (Ref)	–	1.0 (Ref)	–
	Middle third	− 0.04 (− 0.10, 0.03)	0.28	0.04 (− 0.03, 0.11)	0.23
	Highest third	− 0.12 (− 0.19, − 0.05)	< 0.001	− 0.01 (− 0.08, 0.07)	0.84
*AH4*-*I*					
	Lowest third	1.0 (Ref)	–	1.0 (Ref)	–
	Middle third	− 0.06 (− 0.12, 0.00)	0.03	0.01 (− 0.02, 0.05)	0.52
	Highest third	− 0.08 (− 0.13, − 0.02)	0.01	− 0.03 (− 0.07, 0.01)	0.09
*Phonemic fluency*					
	Lowest third	1.0 (Ref)	–	1.0 (Ref)	–
	Middle third	− 0.08 (− 0.15, − 0.01)	0.02	− 0.03 (− 0.09, 0.04)	0.45
	Highest third	− 0.10 (− 0.17, − 0.03)	0.007	− 0.08 (− 0.15, − 0.01)	0.03
*Semantic fluency*					
	Lowest third	1.0 (Ref)	–	1.0 (Ref)	–
	Middle third	− 0.02 (− 0.09, 0.04)	0.45	− 0.05 (− 0.11, 0.00)	0.06
	Highest third	− 0.08 (− 0.14, − 0.01)	0.03	− 0.06 (− 0.13, 0.00)	0.06

^a^Differences are adjusted for age and sex and their interactions with time and time squared and for ethnicity and employment grade and their interactions with time

**Fig. 2:**
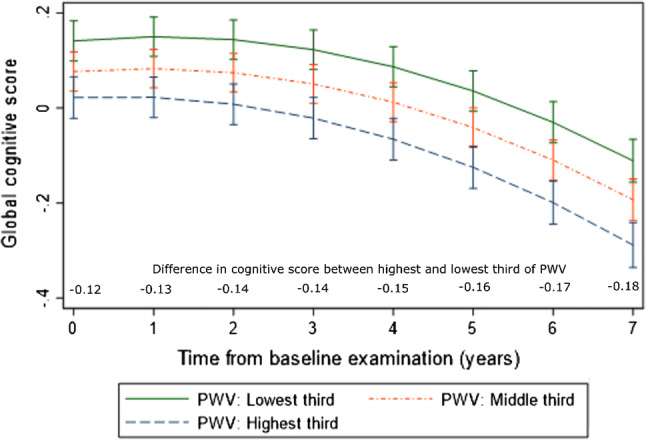
Trajectories (The trajectories of global cognitive score are the predicted values that come from the mixed model that was used to estimate the effects of pulse wave velocity on global cognitive function shown in Table 2) of global cognitive score with follow-up time from baseline examination according to thirds of pulse wave velocity

The declines in global cognitive score with time, after adjustment for sex, ethnicity and employment grade, are shown for the three PWV categories in Fig. 2. The global cognitive scores decrease fastest for those in the highest third of PWV such that the difference between the highest and lowest third of PWV after 7 years is 50% greater than at baseline (− 0.18 vs. − 0.12). We assessed the association between MMSE and PWV, both cross-sectionally and longitudinally and the results were not significant (Supplementary Table 3).

In the analyses to predict those with the fastest cognitive declines as the outcome (binary variable of top 20% vs. rest), individuals in the highest third of PWV were 40% more likely to exhibit rapid declines in cognitive function during prospective follow-up (OR 1.40, 95% CI: 1.13, 1.73), following adjustment for age, sex, ethnicity and employment grade (Table 3). Excluding 4 participants who had prevalent dementia and 10 who had dementia diagnosed during the follow-up made little impact and the coefficient for PWV was reduced by less than 1%. Further sensitivity analyses assessing different percentages (10, 15, 20, and 25%) of participants with the fastest cognitive declines were broadly consistent with the main findings (Supplementary Table 4).

**Table 3:** Association between pulse wave velocity and accelerated global cognitive decline during follow-up

Pulse wave velocity	No. participants	The 20%^a^ of participants with the greatest cognitive decline over the follow-up, N (%)	Odds ratio^b^ (95% CI) *P* value
Lowest third	1316	224 (17.2%)	1.0 (Ref)
Middle third	1300	265 (20.4%)	1.23 (1.01, 1.51) 0.04
Highest third	1212	281 (23.2%)	1.40 (1.13, 1.73) 0.002
Effect per 1 SD increase	3828	770 (20.1%)	1.11 (1.02, 1.21) 0.02

^a^The cut-points for those with the greatest cognitive decline are age, sex and employment grade specific and are based upon the 3828 participants in this analysis

^b^Odds ratios of being in the group with the greatest cognitive decline are adjusted for age, sex, ethnicity and employment grade

## Discussion

Aortic stiffness was associated with faster global cognitive decline over seven years of follow-up. Individuals in the highest third of PWV compared to the lowest had 40% higher odds of cognitive decline. We aimed to identify whether specific cognitive domains contributed to the global decline, but no robust association between arterial stiffness and cognitive decline in all cognitive domains were observed. Decline in phonemic fluency was most clearly associated with aortic stiffness, while decline in reasoning capacity assessed by the AH4-I was linked with aortic stiffness only marginally.

Although several studies [11–20] have identified an inverse association between aortic stiffness and cognition, a few studies [21–23] did not find an association. Our large longitudinal study adds to the evidence that aortic stiffness predicts cognitive decline. Several proposed pathways link aortic stiffness to cognitive decline. First, the stiff aorta loses its cushioning capacity and promotes transmission of potentially deleterious central pressure pulses to the fragile small vessels of target organs such as the brain. Increased pulsatile flow in the brain may accelerate intracranial stenosis, and dysfunction in the cerebral microcirculation [28, 29]. Second, chronic cerebral hypoperfusion may induce cerebral white matter injury or lead to toxic metabolic byproducts accumulation within the brain and blood vessels [30]. Moreover, thrombosis and microinfarcts that can be caused as a result of thickening of the cerebrovascular endothelium and associated endothelial dysfunction are also apparent in malignant hypertension [31]. Third, arterial stiffness is associated with the risk of developing stroke [32] and it has been confirmed that stroke could result in the cognitive impairment [33].

Previously, a study reported concurrent decline in both phonemic and sematic fluency in a group of people with mild cognitive impairment (MCI) [34], while another study [35] showed that individuals with MCI performed worse on phonemic fluency than sematic fluency. While the underlying mechanisms remained largely unknown, evidence shows that multiple brain regions involves during fluency tasks, phonemic fluency tasks are proposed to rely mainly upon executive functioning and pre-frontal lobe processes, whereas sematic fluency tasks are proposed to rely on temporal lobe process [36]. The findings of present study support the hypothesis that aortic stiffness is linked to cognitive decline mediated by frontal lobe atrophy as a result of small vessel disease.

Our findings were in accordance with those from the Health, Aging and Body Composition (Health ABC) study (N = 2488) [17], which found associations between higher arterial stiffness, as measured by PWV, and more rapid cognitive decline, as measured by MMSE over nine years of follow-up. Additionally, previous results from a sub sample (N = 552) [18] of older adults in Health ABC found an association between higher PWV and greater cognitive decline on the psychomotor speed only but not on tests of memory and global cognitive function. Furthermore, findings from Baltimore Longitudinal Study of Aging (BLSA) (N = 582) [19] showed significant associations between higher PWV and more rapid cognitive decline on the Blessed Information Memory Concentration Test (working memory test). However, results from the BLSA study did not provide evidence for an association between PWV and tests of global cognitive function [19]. In contrast to our findings, PWV did not predict cognitive decline between two time points over an average of 5 years in the Rotterdam Study [23].

The inconsistency of results across studies may due to differences in aspects of study design, including the specific neuropsychological tests used, sample size of the study, and differences in participant’s characteristics. Many studies used MMSE to assess cognitive impairments in adults. It has been suggested that MMSE is not a suitable measure for cognitive function among healthy participants due to the ceiling effect. Moreover, MMSE is insensitive to small changes in cognitive function [24]. The sample size is crucial to provide adequate statistical power. Previous studies of the association of arterial stiffness measured by PWV with cognitive decline had fewer than 500 participants.

Our study is the first population-based study to examine the association between arterial stiffness and cognitive decline in a large prospective cohort study of British civil servants who underwent three repeated cognitive assessment over 7 years of follow-up. The PWV is regarded as the gold standard for measuring arterial stiffness and thus provides a great strength to our study. We examined the influence of arterial stiffness on different aspects of cognitive functioning rather than assessing general impairment with a widely used tool for assessing cognitive mental status such as MMSE. A battery of cognitive tests used in this study is a better measure for capturing variability in cognitive scores. The estimates did not change when the analyses were repeated using inverse probability weights showing that our results are less likely to be affected by attrition bias. Our study also has several limitations, the data derived from white-collar civil servants and may not be the representative of general populations. Given the longitudinal nature of the study, mortality or other competing risks may have resulted in an attenuation of our results. Our study has limitations. Cognitive data derived from generally healthy, retired white-collar civil servants may not be broadly representative. Cognitive decline may be slower than among the general population, and this phenomenon may help to explain why we did not observe a clear link between aortic stiffness and change in AH4-I score.

In conclusion, our results indicate that high arterial stiffness is associated with accelerated decline in global cognitive score. Among the domains, decline in phonemic fluency was most clearly linked to stiffness of the aorta. We showed that executive function could be at risk of impairment due to reduced aortic compliance and loss of elasticity. Future studies need to determine if the interventions on reducing or preventing arterial stiffness can be effective in delaying cognitive decline.

## Electronic supplementary material

Below is the link to the electronic supplementary material.

Supplementary material 1 (DOCX 37 kb)
