# Supplementary material for: Association of aortic stiffness with cognitive decline: Whitehall II longitudinal cohort study
Source: Eur J Epidemiol. 2019 Nov 27;35(9):861–9. doi: 10.1007/s10654-019-00586-3 (PMC7441227; doi:10.1007/s10654-019-00586-3)
Supplement: Supplementary file 1 — Supplementary material 1 (DOCX 37 kb) [file 10654_2019_586_MOESM1_ESM.docx]

**Supplementary Online Content**

Association of aortic stiffness with cognitive decline: Whitehall II longitudinal cohort study

**Appendix**

[**Model Specification** 2](#_Toc22557595)

[**Supplementary Table 1** Comparison of characteristics of 6225 participants who attended Phase 9 screening clinic according to whether they are in the study sample 3](#_Toc22557596)

[**Supplementary Table 2** Sensitivity analysis of cross-sectional and longitudinal associations between the thirds of pulse wave velocity and standardized scores of global cognitive score and individual cognitive domains using inverse probability weights. 6](#_Toc22557597)

[**Supplementary Table 3** Parameter estimates for the association between PWV and MMSE (<27 & <26) 8](#_Toc22557598)

[**Supplementary Table 4** Sensitivity analysis for the association between pulse wave velocity and accelerated global cognitive decline during follow-up among 3828 participants 9](#_Toc22557599)

**Model Specification**

The linear mixed effects regression model for cognition, on the i^th^ individual, at the j^th^ occasion, is given by

$${Cognition}_{ij}=\beta_{0}+ \beta_{1}{time}_{ij}+ {\beta_{2}time}_{ij}^{2} +\beta_{3}{pwv2}_{i}+\beta_{4}{pwv3}_{i}+\beta_{5}{pwv2}_{i}{time}_{ij}+\beta_{6}{pwv3}_{i}{time}_{ij}+\beta_{7}X_{1i}+\beta_{8}X_{1i}{time}_{ij}+\beta_{9}X_{1i}{time}_{ij}^{2} +\beta_{10}X_{2i}+\beta_{11}X_{2i}{time}_{ij}+U_{0i}+ U_{1i}{time}_{ij}+ e_{ij}$$

where *Cognition_ij_* is the cognitive score of the i^th^ participant at the j^th^ occasion, *time_ij_* is the delay since the PWV measurement at 2007/09 for the i^th^ participant at the j^th^ occasion, *pwv2_i_* and *pwv3_i_* are indicator variables for the middle and highest third of PWV in 2007/09 of the i^th^ participant, *X_1i_* is a vector of covariates (age, sex, age by sex interaction) for the i^th^ participant, *X_2i_* is a vector of covariates (ethnicity, employment grade) for the i^th^ participant. U_0i_ is the random intercept, U_1i_ is the random slope, and e_ij_ is the residual. In the present data, cognitive score is measured on three occasions (j = 1, 2, 3).

In this model, the trajectory of cognitive function over time is quadratic and is described by the coefficients, *β_0_* (intercept) and *β_1_* and *β_2_* (slope terms). The coefficient vectors, *β_7_*, *β_8_* and *β_9_*, adjust the PWV effects on cognition for age and sex by allowing the quadratic trajectories of cognitive score to differ by age in men and women separately. The coefficients *β_3_* and *β_4_* show the cross-sectional association of cognitive function with the middle and highest thirds of PWV at 2007/09. Coefficients *β_5_* and *β_6_* show how change in cognitive function over time is modified by an individual being in the middle or highest third of PWV, compared to the lowest third. The difference in cognitive function between the highest and lowest thirds of PWV is *β_4_* at the time of the PWV measurement in 2007/09, and changes to (*β_4_* + *β_6_*T*)* after time T. Lastly, the coefficient vector, *β_10_*, shows the cross-sectional associations of cognition with ethnicity and employment grade while the coefficient vector, *β_11_*, shows how the change in cognitive function over time is modified by ethnicity and employment grade.

# Supplementary Table 1 Comparison of characteristics of 6225 participants who attended Phase 9 screening clinic according to whether they are in the study sample

|  | Study sample | | *P* value |
| --- | --- | --- | --- |
|  | Excluded | Included |  |
|  | [Mean (SD) or %] | [Mean (SD) or %] |  |
|  |  |  |  |
| Number | 1925 | 4300 |  |
|  |  |  |  |
| Age at Phase 9 clinic, y | 67.1 (6.2) | 65.3 (5.7) | <0.001 |
|  |  |  |  |
| Female, % | 36.1 | 25.3 | <0.001 |
| Ethnicity |  |  | 0.004 |
| - *White* | 91.8 | 92.3 |  |
| - *Non-White* | 8.2 | 7.7 |  |
| BMI (kg/m^2^) |  |  | 0.46 |
| - *Underweight (<18.5)* | 1.2 | 0.9 |  |
| - *Normal weight (18.5 - 24.9)* | 28.1 | 40.2 |  |
| - *Overweight (25.0 – 29.9)* | 39.7 | 44.4 |  |
| - *Obese (≥ 30.0)* | 31.1 | 14.5 |  |
| Systolic Blood Pressure (mmHg) | 128.0 (17.6) | 124.5 (15.5) | <0.001 |
| Diastolic Blood Pressure (mmHg) | 72.2 (10.7) | 70.7 (10.0) | <0.001 |
| Anti-hypertension medication, % | 45.0 | 32.7 | <0.001 |
| Lipid lowering medication, % | 39.8 | 30.6 | <0.001 |
|  |  |  |  |
| Education |  |  | <0.001 |
| - *≤ Lower secondary* | 40.5 | 31.0 |  |
| - *Higher secondary* | 27.1 | 28.1 |  |
| - *≥ Degree* | 32.5 | 39.9 |  |
| Employment grade |  |  | <0.001 |
| - *High* | 42.3 | 49.8 |  |
| - *Intermediate* | 43.6 | 41.6 |  |
| - *Low* | 14.1 | 8.6 |  |
| Smoking habit |  |  | 0.002 |
| - *Never* | 45.5 | 49.1 |  |
| - *Ex-smoker* | 47.6 | 46.0 |  |
| - *Current* | 6.9 | 5.0 |  |
| Alcohol consumption |  |  | <0.001 |
| - *No alcohol* | 24.4 | 16.4 |  |
| - *Moderate alcohol* | 60.6 | 67.1 |  |
| - *Heavy alcohol* | 15.0 | 16.5 |  |
| Moderate or vigorous physical activity |  |  | <0.001 |
| - *< 1 hr/wk* | 27.4 | 21.9 |  |
| - *1 - 6.9 hrs/wk* | 58.6 | 60.9 |  |
| - *≥ 7 hrs/wk* | 13.9 | 17.2 |  |
|  |  |  |  |
| Diabetes, % | 16.8 | 11.1 | <0.001 |
| History of CVD, % | 19.7 | 11.7 | <0.001 |

**Supplementary Table 2** Sensitivity analysis of cross-sectional and longitudinal associations between the thirds of pulse wave velocity and standardized scores of global cognitive score and individual cognitive domains using inverse probability weights.

| Cognitive domain outcome | Pulse wave velocity |  | Standardized cognitive score at baseline | |  | Change in standardized cognitive score (per 7 years) | |
| --- | --- | --- | --- | --- | --- | --- | --- |
|  |  |  | Difference* (95% CI) | *P* value |  | Difference* (95% CI) | *P* value |
|  |  |  |  |  |  |  |  |
| Global cognitive score |  |  |  |  |  |  |  |
|  | Lowest third |  | 1.0 (Ref) | - |  | 1.0 (Ref) | - |
|  | Middle third |  | -0.08 (-0.14, -0.02) | 0.01 |  | -0.04 (-0.09, 0.00) | 0.06 |
|  | Highest third |  | -0.14 (-0.20, -0.07) | <0.001 |  | -0.05 (-0.10, 0.00) | 0.06 |
| Memory |  |  |  |  |  |  |  |
|  | Lowest third |  | 1.0 (Ref) | - |  | 1.0 (Ref) | - |
|  | Middle third |  | -0.04 (-0.11, 0.02) | 0.19 |  | 0.01 (-0.05, 0.08) | 0.71 |
|  | Highest third |  | -0.13 (-0.20, -0.06) | <0.001 |  | 0.00 (-0.06, 0.09) | 0.72 |
| AH4-I |  |  |  |  |  |  |  |
|  | Lowest third |  | 1.0 (Ref) | - |  | 1.0 (Ref) | - |
|  | Middle third |  | -0.08 (-0.14, -0.02) | 0.001 |  | -0.01 (-0.04, 0.03) | 0.75 |
|  | Highest third |  | -0.10 (-0.16, -0.03) | 0.003 |  | -0.04 (-0.08, 0.00) | 0.06 |
| Phonemic fluency |  |  |  |  |  |  |  |
|  | Lowest third |  | 1.0 (Ref) | - |  | 1.0 (Ref) | - |
|  | Middle third |  | -0.08 (-0.15, -0.02) | 0.01 |  | -0.06 (-0.12, 0.01) | 0.10 |
|  | Highest third |  | -0.12 (-0.19, -0.05) | 0.001 |  | -0.08 (-0.16, 0.00) | 0.04 |
| Semantic fluency |  |  |  |  |  |  |  |
|  | Lowest third |  | 1.0 (Ref) | - |  | 1.0 (Ref) | - |
|  | Middle third |  | -0.02 (-0.09, 0.04) | 0.50 |  | -0.08 (-0.14, -0.01) | 0.02 |
|  | Highest third |  | -0.08 (-0.15, -0.01) | 0.02 |  | -0.05 (-0.12, 0.02) | 0.13 |
|  |  |  |  |  |  |  |  |

*Differences are adjusted for age and sex and their interactions with time and time squared and for ethnicity and employment grade and their interactions with time.

**Supplementary Table 3** Parameter estimates for the association between PWV and MMSE (<27 & <26)

| Using MMSE <27 | (1068 cases with MMSE <27,  out of 11447 records across all three phases) | | |
| --- | --- | --- | --- |
|  | Cross-sectional association | | |
| **Pulse wave velocity^a^** | **Coefficient^b^** | **Standard Error** | ***P* value** |
| Middle third | 0.08 | 0.17 | 0.63 |
| Highest third | 0.09 | 0.17 | 0.58 |
|  | Longitudinal association | | |
| Middle third | 0.20 | 0.23 | 0.36 |
| Highest third | 0.17 | 0.23 | 0.47 |
|  |  |  |  |
| Using MMSE <26 | (491 cases with MMSE <26,  out of 11447 records across all three phases) | | |
|  | Cross-sectional association | | |
| **Pulse wave velocity^a^** | **Coefficient^b^** | **Standard Error** | ***P* value** |
| Middle third | -0.15 | 0.26 | 0.58 |
| Highest third | 0.16 | 0.25 | 0.50 |
|  | Longitudinal association | | |
| Middle third | 0.51 | 0.35 | 0.14 |
| Highest third | 0.17 | 0.33 | 0.61 |
| ^a^ Lowest third of PWV is the reference group  ^b^ Coefficients are from logistic mixed models and are adjusted for age, sex, ethnicity and employment grade and their interactions with time | | | |

# Supplementary Table 4 Sensitivity analysis for the association between pulse wave velocity and accelerated global cognitive decline during follow-up among 3828 participants

| **Outcome definition - Percentage of participants with the greatest cognitive decline over the follow-up^a^** |  | **Number of participants with the greatest cognitive decline over the follow-up** | **Odds ratio^b^ (95% CI)** | ***P* value** |
| --- | --- | --- | --- | --- |
|  |  |  |  |  |
| 10% |  | 386 | 1.14 (1.02, 1.28) | 0.02 |
| 15% |  | 577 | 1.10 (1.00, 1.21) | 0.05 |
| 20% |  | 770 | 1.11 (1.02, 1.21) | 0.02 |
| 25% |  | 958 | 1.14 (1.05, 1.24) | 0.001 |
|  |  |  |  |  |

^a^ The cut-points for those with the greatest cognitive decline are age, sex and employment grade specific and are based upon the 3828 participants in this analysis

^b^ Odds ratio of being in the group with the greatest cognitive decline associated with an increase of 1SD in pulse wave velocity adjusted for age, sex, ethnicity and employment grade
